# Laparoscopic Endometriosis Phenotypes and Preoperative Non-suspicion of Endometriosis in Women With Superficial Disease: A Retrospective Study in Kinshasa

**DOI:** 10.7759/cureus.118310

**Published:** 2026-10-21

**Authors:** Guy Lambert M Monzango Sibo, Laetitia Itewa Monka

**Affiliations:** 1 Gynecology, University of Kinshasa, Kinshasa, COD; 2 Gynecology and Obstetrics, Cliniques Universitaires de Kinshasa, Kinshasa, COD

**Keywords:** deep dyspareunia, endometrioma, infertility, kinshasa, laparoscopy, preoperative recognition, superficial peritoneal endometriosis

## Abstract

Background

Superficial peritoneal endometriosis (SPE) is difficult to identify before surgery, especially without concomitant ovarian or deep disease. We described laparoscopic phenotypes and examined factors associated with the absence of documented preoperative suspicion of any endometriosis among women subsequently found to have SPE.

Methods

We retrospectively analyzed gynecologic laparoscopies performed at three Kinshasa sites between January 2019 and June 2026 by one surgeon. Superficial, ovarian, and deep phenotypes were recorded as non-mutually exclusive. The analytical outcome was absence of documented preoperative suspicion of endometriosis of any phenotype among women with laparoscopically identified SPE and classifiable preoperative records. Modified Poisson models with robust variance estimated exploratory adjusted prevalence ratios (aPRs).

Results

Of 1,743 gynecologic laparoscopies, 216 (12.4%; 95% CI, 10.9%-14.0%) showed endometriosis and 1,527 (87.6%) did not. SPE occurred in 139/216 (64.4%), ovarian disease in 137/216 (63.4%), and deep disease in 68/216 (31.5%); 104/216 (48.1%) had overlapping phenotypes. Among 135 women with SPE whose preoperative records were classifiable, 83 (61.5%; 95% CI, 53.1%-69.3%) lacked documented suspicion of endometriosis; this proportion was 46/56 (82.1%) in isolated SPE. In the clinical model, infertility was associated with higher non-suspicion (aPR 1.59; 95% CI, 1.07-2.35), while deep dyspareunia was associated with lower non-suspicion (aPR 0.65; 95% CI, 0.45-0.94). With additional adjustment for coexisting operative phenotypes, the infertility association weakened (aPR 1.39; 95% CI, 0.95-2.04) and the dyspareunia estimate remained borderline (aPR 0.70; 95% CI, 0.49-1.00).

Conclusions

SPE and ovarian disease were the most common laparoscopic phenotypes in this selected surgical cohort. An absence of documented suspicion of any endometriosis was frequent among women with SPE, especially isolated SPE. Associations with infertility and deep dyspareunia are exploratory and may reflect clinical documentation, surgical referral pathways, and coexisting disease. Prospective standardized assessments are needed.

## Introduction

Endometriosis affects an estimated 10% of women of reproductive age and is associated with pelvic pain, dysmenorrhea, dyspareunia, infertility, and impaired quality of life [1]. Its anatomical phenotypes include superficial peritoneal endometriosis (SPE), ovarian endometrioma, and deep endometriosis [2]. Because these phenotypes may coexist, their distribution and overlap are relevant to interpreting preoperative clinical suspicion.

Although expert ultrasonography and magnetic resonance imaging can identify many ovarian and deep lesions, SPE remains more difficult to recognize. The European Society of Human Reproduction and Embryology (ESHRE) emphasizes that negative imaging does not exclude endometriosis, particularly SPE [3]. The 2025 International Deep Endometriosis Analysis (IDEA) addendum proposes standardized sonographic assessment of superficial lesions [4], but the frequency of documented preoperative suspicion in routine surgical care remains poorly characterized.

Symptoms do not resolve this uncertainty. In a 2026 multicenter study of symptomatic women with negative examination and imaging, a symptom-based model for isolated SPE performed poorly in external validation (area under the curve, 0.49) [5]. However, predicting SPE rather than no endometriosis differs from assessing whether endometriosis of any phenotype had been suspected before laparoscopy in women subsequently found to have SPE. Coexisting ovarian or deep lesions may influence this recognition.

This distinction is relevant in Kinshasa, where phenotype-specific evidence is limited. A previous study of 80 women with laparoscopically and histologically confirmed endometriosis reported a mean diagnostic delay of approximately 12 years [6] but did not describe phenotype overlap across a broader laparoscopic series or document preoperative suspicion among women with SPE. Consequently, it remains unclear whether isolated SPE differs from overlapping phenotypes in its frequency of documented preoperative non-suspicion.

The central problem is therefore not solely whether imaging can detect superficial lesions, but whether any endometriosis was documented as suspected before surgery in women with subsequently identified SPE. Absence of documented suspicion is a record-based outcome, not proof that clinicians failed to consider endometriosis or a measure of the sensitivity of clinical assessment or imaging.

The primary objective was to describe the distribution and coexistence of superficial, ovarian, and deep endometriosis identified during gynecologic laparoscopy in Kinshasa. Secondary objectives were to estimate the proportion of laparoscopies with identified endometriosis and, among women with SPE, to quantify the absence of documented preoperative suspicion of endometriosis of any phenotype and explore associated clinical characteristics.

## Materials and methods

Study design and setting

We conducted a retrospective three-site analytical observational study of women undergoing gynecologic laparoscopy between January 1, 2019, and June 30, 2026, at Onyx Medical Center (OMC), Clinique Présidentielle de l’Union Africaine (CPUA), and Cliniques Universitaires de Kinshasa (CUK), Kinshasa, Democratic Republic of the Congo.

All included laparoscopies were performed by the same experienced gynecologic laparoscopic surgeon using a consistent approach to pelvic exploration and operative documentation. Operative reports were systematically completed, laparoscopic photographs and/or videos were archived, and preoperative ultrasound images were retained when available. The study was reported in accordance with Strengthening the Reporting of Observational Studies in Epidemiology (STROBE) recommendations [7].

Study population and analytical populations

The source cohort comprised all 1,743 gynecologic laparoscopies performed at the three participating centers during the study period. No gynecologic laparoscopic procedure was excluded from the source cohort before analysis.

Three nested analytical populations were defined: (1) all 1,743 gynecologic laparoscopies, including 216 with and 1,527 without laparoscopically identified endometriosis; (2) the 216 women with laparoscopically identified endometriosis, used to characterize anatomical phenotypes; and (3) the 139 women with SPE, of whom 135 had classifiable preoperative suspicion status and were included in the suspected-versus-unsuspected comparison. The 1,527 procedures without endometriosis were not excluded. The four women with indeterminate preoperative documentation were retained in the descriptive analyses of endometriosis and its phenotypes but not included in the comparison by suspicion status.

Eligibility criteria and data sources

The study scope comprised gynecologic laparoscopies performed at the participating centers between January 1, 2019, and June 30, 2026. All 1,743 procedures in this defined source population were included, with zero exclusions. Procedures performed exclusively by laparotomy and operations for non-gynecologic indications were outside the study scope rather than exclusions from the laparoscopic cohort.

Data were extracted from operative registers, clinical records, preoperative consultations, ultrasound and magnetic resonance imaging reports when available, archived ultrasound images, operative reports, laparoscopic photographs/videos, and histopathology reports.

Preoperative variables and suspicion status

Preoperative variables included age, body mass index, infertility, dysmenorrhea, severe dysmenorrhea, pain duration longer than three years, chronic pelvic pain, deep dyspareunia, dyschezia, cyclical urinary symptoms, previous pelvic surgery, and the principal surgical indication. Infertility status was taken from the documented clinical variable and was not inferred solely from the operative indication.

Preoperative suspicion was assessed exclusively from contemporaneous information recorded before surgery. A woman was “suspected” when the record explicitly documented clinical suspicion or previously known endometriosis, an endometrioma, suspected deep disease, or a positive preoperative ultrasound assessment of any endometriosis phenotype. A woman was “not documented as suspected” if her available record was considered classifiable and none of those indications were recorded. This definition concerns suspicion of endometriosis generally, not specific preoperative recognition of superficial lesions. Cases without sufficiently interpretable documentation were marked indeterminate rather than presumed negative. In a retrospective record review, absence of documentation cannot be equated with proof that a clinician did not consider the diagnosis.

In the harmonized analysis, a positive contemporaneous ultrasound report overrode a raw database label of “unsuspected”; the raw database classification was examined in a sensitivity analysis. The study did not formally measure diagnostic sensitivity, and retrospective review of archived ultrasound images was not used to redefine what the treating clinician knew before laparoscopy.

Laparoscopic diagnosis and phenotype definitions

Endometriosis was classified as laparoscopically identified when operative documentation described one or more macroscopically compatible lesions. Photographs or videos were available to corroborate operative descriptions. Histopathological confirmation was recorded separately; absence of tissue confirmation did not automatically exclude macroscopically suspected disease. This approach may lead to phenotype misclassification, particularly for superficial lesions.

Phenotypes were defined according to international terminology [2]. SPE comprised lesions involving the peritoneal surface without operative evidence of deep infiltration. Ovarian endometriosis was defined by one or more laparoscopically identified endometriomas. Deep endometriosis was defined by deep, nodular, or infiltrative disease involving structures such as the uterosacral ligaments/torus, retrocervical region, rectovaginal compartment, rectosigmoid, bladder, or ureter.

The three phenotypes were recorded independently and were not treated as mutually exclusive. Combinations were subsequently derived as isolated superficial, isolated ovarian, isolated deep, superficial plus ovarian, superficial plus deep, ovarian plus deep, and simultaneous superficial, ovarian, and deep disease.

Ultrasound archives and the IDEA 2025 framework

Archived preoperative ultrasound images were available for most women with endometriosis. However, the complete 2025 IDEA protocol for SPE was not retrospectively reconstructed because examinations performed before publication of the consensus had not systematically captured all dynamic components, including site-specific tenderness, ovarian mobility, organ sliding, and standardized real-time scanning maneuvers [4]. The original contemporaneous ultrasound interpretation, rather than retrospective reinterpretation after knowledge of surgery, was used to define preoperative suspicion.

Outcomes

The principal descriptive outcome was anatomical phenotype distribution. The analytic outcome was absence of documented preoperative suspicion of endometriosis of any phenotype among women with SPE and classifiable records; it was not a measure of diagnostic sensitivity for SPE specifically. Secondary outcomes included overlapping phenotypes, lesion sites, histological confirmation, and exploratory associations between documented clinical characteristics and non-suspicion.

Statistical analysis

Analyses were performed using Python 3.13.5 (Python Software Foundation, Wilmington, DE) with Statsmodels 0.14.6, SciPy 1.17.0, NumPy 2.3.5, and pandas 2.2.3. Continuous variables were summarized as mean ± standard deviation and categorical variables as counts and percentages. Proportions were accompanied by 95% confidence intervals.

Continuous characteristics were compared using Student’s t-test and categorical characteristics using chi-square or Fisher’s exact tests, as applicable. Given the common binary outcome, modified Poisson regression with robust variance was used to estimate adjusted prevalence ratios (APRs) and 95% confidence intervals [8]. Associations were interpreted within this selected surgical cohort rather than causally.

The primary clinical model included age, infertility, severe dysmenorrhea, pain duration over three years, deep dyspareunia, and dyschezia, selected for clinical relevance and preoperative availability rather than univariable significance. Coexisting operative phenotypes were omitted from the primary clinical model because they were not established preoperatively; an extended explanatory model included ovarian and deep disease. Interpretation must account for possible collinearity, documentation bias, and referral/surgical-indication selection.

Reported sensitivity analyses used (1) unharmonized database suspicion status, (2) histologically confirmed SPE, (3) availability of operative video, and (4) additional adjustment for ovarian and deep phenotypes. The manuscript reports a two-sided significance threshold of 0.05; near-threshold estimates should be interpreted by effect size and interval precision rather than as binary evidence.

Data validation and missingness

All 1,743 gynecologic laparoscopies in the source cohort were included without exclusions: 216 showed laparoscopically identified endometriosis, and 1,527 did not. Among the 139 women with SPE, four had indeterminate preoperative suspicion status; these women remained in the descriptive cohort, while 135 classifiable records contributed to the comparison of preoperative suspicion. The six covariates used in the primary regression model were complete in those 135 records. Descriptive and regression analyses were performed using the analytical dataset; primary patient records were not independently reabstracted for this analysis.

Ethical considerations

The study was approved by the Ethics Committee of the School of Public Health, University of Kinshasa (approval number ESP/CE/769/2026). The analysis used retrospective clinical and operative data.

## Results

Study population and frequency of endometriosis

All 1,743 gynecologic laparoscopies performed at the three centers during the study period were included, with no procedures excluded before analysis. Endometriosis was identified in 216 (12.4%; 95% CI, 10.9%-14.0%), while the other 1,527 procedures (87.6%) showed no laparoscopically identified endometriosis. OMC contributed 1,275 laparoscopies, including 158 endometriosis cases (12.4%), and CPUA contributed 464 laparoscopies, including 57 cases (12.3%); these proportions did not differ (p = 0.952). CUK contributed four laparoscopies, including one case; no center-specific inference was made because of this very small denominator.

There was no statistically significant temporal change in the probability of identifying endometriosis across the study period (OR per calendar year 1.06; 95% CI, 0.99-1.13; p = 0.108).

Patient characteristics and the principal surgical indications are presented in Table 1.

**Table 1:** Clinical characteristics and principal surgical indications among women with laparoscopically identified endometriosis (n = 216)

Characteristic	n (%) or mean ± SD
Age, years	31.7 ± 6.2
Body mass index, kg/m²	25.7 ± 3.9
Infertility	140 (64.8%)
Dysmenorrhea	177 (81.9%)
Severe dysmenorrhea	99 (45.8%)
Chronic pelvic pain	85 (39.4%)
Deep dyspareunia	71 (32.9%)
Dyschezia	28 (13.0%)
Cyclical urinary symptoms	22 (10.2%)
Previous pelvic surgery	48 (22.2%)
Infertility/tuboperitoneal assessment	72 (33.3%)
Adnexal cyst or mass	59 (27.3%)
Pelvic pain	51 (23.6%)
Hydrosalpinx/other tubal disease	19 (8.8%)
Uterine leiomyoma	8 (3.7%)
Other gynecologic indication	4 (1.9%)
Ectopic pregnancy	3 (1.4%)

Distribution of laparoscopic phenotypes

SPE was identified in 139/216 women (64.4%; 95% CI, 57.8%-70.4%), ovarian endometriosis in 137/216 (63.4%; 95% CI, 56.8%-69.6%), and deep endometriosis in 68/216 (31.5%; 95% CI, 25.7%-38.0%). Because phenotypes could coexist, individual phenotype percentages were not mutually exclusive.

At least two phenotypes were present in 104/216 women (48.1%).

The distribution and overlap of anatomical phenotypes are summarized in Table 2.

**Table 2:** Distribution and overlap of laparoscopic endometriosis phenotypes The first three phenotype categories are non-mutually exclusive; the seven phenotype-combination categories are mutually exclusive.

Phenotype	n (%)
Superficial peritoneal endometriosis	139 (64.4%)
Ovarian endometriosis	137 (63.4%)
Deep endometriosis	68 (31.5%)
Isolated superficial	58 (26.9%)
Isolated ovarian	46 (21.3%)
Isolated deep	8 (3.7%)
Superficial + ovarian	44 (20.4%)
Superficial + deep	13 (6.0%)
Ovarian + deep	23 (10.6%)
Superficial + ovarian + deep	24 (11.1%)
≥2 phenotypes	104 (48.1%)

The posterior peritoneum was involved in 111/216 women (51.4%), followed by the left ovary in 90 (41.7%), anterior peritoneum in 82 (38.0%), right ovary in 78 (36.1%), and uterosacral ligament/torus region in 53 (24.5%). Retrocervical, rectosigmoid, and bladder involvement were documented in 20 (9.3%), 18 (8.3%), and six women (2.8%), respectively.

Operative video was available in 212/216 cases (98.1%) and archived preoperative ultrasound images in 200/216 (92.6%). Biopsy or excision was performed in 182 women; histology confirmed endometriosis in 166/182 sampled cases (91.2%), representing 76.9% of all laparoscopically identified cases.

Preoperative non-suspicion of superficial peritoneal endometriosis

Among 139 women with laparoscopically identified SPE, preoperative documentation was classifiable in 135: 83 had no documented suspicion of endometriosis of any phenotype, 52 had documented suspicion, and four were indeterminate. Thus, non-suspicion was recorded in 83/135 (61.5%; 95% CI, 53.1%-69.3%). This is a measure of documentation-defined recognition among operated patients, not the false-negative rate of clinical examination or imaging.

Non-suspicion varied across phenotype combinations (reported p < 0.001): 46/56 (82.1%) in isolated SPE, 24/44 (54.5%) in superficial-plus-ovarian disease, 5/13 (38.5%) in superficial-plus-deep disease, and 8/22 (36.4%) when all three phenotypes coexisted. The denominator of 56 isolated cases reflects exclusion of two indeterminate records from 58 laparoscopically identified isolated SPE cases; the other two indeterminate records were in the triple-phenotype category (24 total, 22 classifiable). The crude reported prevalence ratio for isolated versus coexisting SPE was 1.75 (95% CI, 1.35-2.29; p < 0.001). These contrasts partly reflect the outcome definition: ovarian or deep lesions can themselves prompt suspicion of endometriosis.

Clinical characteristics associated with preoperative non-suspicion

Preoperative clinical characteristics by documented suspicion status are compared in Table 3.

**Table 3:** Preoperative characteristics according to suspicion status among women with superficial peritoneal endometriosis Values are n (%) unless otherwise indicated.

Variable	Unsuspected (n = 83)	Suspected (n = 52)	p-value
Age, years	30.9 ± 5.9	32.3 ± 6.0	0.212
Body mass index, kg/m²	25.7 ± 4.2	25.9 ± 3.8	0.698
Infertility	67 (80.7%)	30 (57.7%)	0.004
Severe dysmenorrhea	37 (44.6%)	25 (48.1%)	0.691
Pain duration >3 years	34 (41.0%)	23 (44.2%)	0.708
Chronic pelvic pain	28 (33.7%)	23 (44.2%)	0.221
Deep dyspareunia	18 (21.7%)	24 (46.2%)	0.003
Dyschezia	10 (12.0%)	9 (17.3%)	0.392
Cyclical urinary symptoms	11 (13.3%)	3 (5.8%)	0.165
Previous pelvic surgery	13 (15.7%)	12 (23.1%)	0.28

Infertility was more frequent among women with unsuspected SPE than among women with suspected SPE (80.7% vs. 57.7%; p = 0.004), whereas deep dyspareunia was less frequent (21.7% vs. 46.2%; p = 0.003). No statistically significant differences were observed for age, body mass index, severe dysmenorrhea, symptom duration longer than three years, chronic pelvic pain, dyschezia, cyclical urinary symptoms, or previous pelvic surgery.

Multivariable analysis

The primary multivariable estimates are presented in Table 4.

**Table 4:** Primary modified Poisson regression for absence of documented preoperative suspicion of endometriosis among women with superficial peritoneal disease This exploratory model included only recorded preoperative clinical variables. The endpoint was absence of documented suspicion of any endometriosis among women with SPE, not specific non-detection of SPE. aPR: adjusted prevalence ratio; CI: confidence interval

Variable	aPR	95% CI	p-value
Age, per year	0.99	0.97-1.01	0.22
Infertility	1.59	1.07-2.35	0.022
Severe dysmenorrhea	0.94	0.73-1.21	0.618
Pain duration >3 years	0.93	0.72-1.20	0.572
Deep dyspareunia	0.65	0.45-0.94	0.022
Dyschezia	0.91	0.57-1.43	0.673

In the primary clinical model, infertility was associated with more frequent documentation-defined non-suspicion (aPR 1.59; 95% CI, 1.07-2.35; p = 0.022) and deep dyspareunia with less frequent non-suspicion (aPR 0.65; 95% CI, 0.45-0.94; p = 0.022). These estimates are exploratory and are not evidence that infertility causes missed diagnosis or that dyspareunia increases test accuracy.

Sensitivity analyses

The sensitivity analysis estimates are presented in Table 5.

**Table 5:** Sensitivity analyses for the two principal clinical associations aPR: adjusted prevalence ratio; SPE: superficial peritoneal endometriosis

Analysis	Infertility, aPR (95% CI); p	Deep dyspareunia, aPR (95% CI); p
Primary harmonized status (n = 135)	1.59 (1.07-2.35); p = 0.022	0.65 (0.45-0.94); p = 0.022
Raw database status (n = 134)	1.71 (1.16-2.52); p = 0.007	0.69 (0.49-0.96); p = 0.028
Histologically confirmed SPE (n = 102)	1.61 (1.06-2.44); p = 0.026	0.69 (0.47-1.00); p = 0.050
Operative video available (n = 132)	1.65 (1.09-2.49); p = 0.017	0.68 (0.47-0.98); p = 0.037
Additional adjustment for ovarian/deep phenotype (n = 135)	1.39 (0.95-2.04); p = 0.091	0.70 (0.49-1.00); p = 0.047

The infertility association was similar in analyses using raw database labels and restricted to histologically confirmed or video-documented SPE; it attenuated after adjustment for coexisting ovarian/deep disease (aPR 1.39; 95% CI, 0.95-2.04; p = 0.091). In the extended model, deep dyspareunia had a near-threshold estimate (aPR 0.70; 95% CI, 0.49-1.00 after rounding; p = 0.047). Coexisting deep disease was associated with less frequent documented non-suspicion (aPR 0.60; 95% CI, 0.39-0.92; p = 0.020), although this association may partly reflect the operational definition of clinical suspicion.

There was no statistically significant temporal trend in SPE non-suspicion over the study period (prevalence ratio per calendar year 0.97; 95% CI, 0.91-1.03; p = 0.279). 

## Discussion

This surgical cohort describes the overlap of laparoscopically identified phenotypes in Kinshasa. Endometriosis was identified in approximately one of eight eligible laparoscopies, and nearly half of the identified cases exhibited multiple phenotypes. Among women with SPE and classifiable records, 61.5% had no documented preoperative suspicion of endometriosis of any phenotype. Non-suspicion was especially frequent with isolated superficial disease. These findings describe recognition documented within a selected operative pathway, not population prevalence or imaging sensitivity.

The 12.4% proportion should not be interpreted as the population prevalence of endometriosis in Kinshasa. The denominator consisted of women selected for gynecologic laparoscopy and therefore reflects surgical indications, referral pathways, and access to minimally invasive surgery. Nevertheless, the almost identical proportions observed at OMC and CPUA and the absence of a significant temporal trend suggest that the overall estimate was not driven by a single high-volume period or by one of the two principal sites.

The phenotype distribution reinforces the anatomical heterogeneity of endometriosis. International terminology treats superficial, ovarian, and deep disease as distinct but potentially coexisting forms [2]. In our series, 48.1% of women had at least two phenotypes. This is consistent with prospective data demonstrating substantial coexistence of lesion types among women undergoing surgery for endometriosis-associated infertility [9]. Recording each phenotype independently therefore provides more informative anatomical characterization than forcing every patient into a single mutually exclusive category.

Documented preoperative suspicion was less frequent in women with isolated SPE than in those with coexisting ovarian or deep disease. This is compatible with the recognized imaging challenges of superficial lesions [3,4], but the study did not apply a standardized preoperative imaging protocol and cannot estimate the performance of ultrasonography. Furthermore, the endpoint deliberately counts suspicion of an endometrioma or deep endometriosis as suspicion of endometriosis, whether or not a superficial lesion was separately recognized. Hence, differences by phenotype combination may partly arise from the classification rule itself.

Infertility was more common in women without documented preoperative suspicion (80.7%) than in those with documented suspicion (57.7%). The clinical model indicated an association, but operated infertility patients may reach laparoscopy for tuboperitoneal investigations without a recorded endometriosis hypothesis. Consequently, referral pathways and surgery indication are plausible sources of selection bias; infertility should not be regarded as an independently validated diagnostic marker for SPE.

An association between superficial endometriosis and infertility is biologically and clinically plausible. Reis et al. compared 203 women with isolated histologically confirmed SPE with 1,292 endometriosis-free controls and found an independent association with primary infertility, as well as dysmenorrhea and deep dyspareunia [10]. Their outcome, however, was the presence of SPE, whereas our outcome was preoperative recognition among women who already had SPE. These questions are related but not equivalent.

The association of infertility weakened when coexisting ovarian and deep phenotypes were included. This extended adjustment is explanatory and does not identify a causal pathway because operative anatomy and preoperative recognition are linked by the outcome definition, and selection into surgery may depend on symptoms and imaging. The numerical association should therefore be presented as hypothesis-generating rather than robust evidence of an independent effect.

Deep dyspareunia was less frequently documented among women without preoperative suspicion and showed an exploratory association with greater likelihood of documented recognition. Clinicians may be more inclined to consider endometriosis when dyspareunia is recorded, but different elicitation or recording of symptoms could generate this pattern. The extended estimate (aPR 0.70, p = 0.047) is close to the conventional threshold, and the confidence interval rounds to 1.00, requiring cautious interpretation.

Our results also differ in an informative way from the 2026 study by Reiser et al. [5]. In that study, severe dysmenorrhea and pain lasting more than three years were associated with isolated SPE in women with negative examination and imaging, but the prediction model performed poorly during external validation. In our analysis, neither severe dysmenorrhea nor prolonged pain duration was independently associated with preoperative non-suspicion. This does not represent failed replication because the outcomes are different: Reiser et al. attempted to distinguish SPE from no endometriosis, whereas we compared recognized with unrecognized disease among women with SPE. Together, the studies argue against relying on a single symptom profile to identify superficial disease.

The findings are relevant to the local diagnostic context. A previous Kinshasa study documented an average diagnostic delay of approximately 12 years among women with surgically and histologically confirmed endometriosis [6]. The present study extends that observation by identifying isolated superficial disease as a phenotype particularly vulnerable to preoperative non-recognition. Improving detection in this setting will likely require standardized history-taking, awareness of infertility-associated presentations, and dedicated ultrasound approaches rather than symptom screening alone.

This study has several strengths. The denominator comprised 1,743 gynecologic laparoscopies over more than seven years. All procedures were performed by the same experienced surgeon, reducing inter-surgeon variability in pelvic exploration and recognition of lesions. Operative videos were available for 98.1% of endometriosis cases and archived ultrasound images for 92.6%. Phenotypes were analyzed as potentially coexisting rather than mutually exclusive. Finally, the principal associations were examined across several sensitivity analyses, including histologically confirmed and video-documented subsets.

Important limitations include the retrospective, documentation-dependent definition of suspicion, unavailable information on assessor masking and documentation-completeness rules, and the possibility that absence of a chart entry does not indicate absence of clinical suspicion. The endpoint refers to suspicion of any endometriosis among women with SPE, not to recognition of the superficial phenotype specifically. Referral pathways and surgical indications may have introduced selection bias, and findings cannot be generalized to the population of Kinshasa. Not all macroscopic lesions underwent histopathological confirmation, and blinded independent video review was not performed. Almost all procedures came from two centers, while the third contributed four; all were performed by a single surgeon. The full IDEA 2025 dynamic ultrasound protocol was not used systematically during the study period. The near-threshold finding for dyspareunia and attenuation of the infertility association after phenotype adjustment discourage causal or diagnostic interpretations.

The present data should therefore be considered hypothesis-generating rather than sufficient for a diagnostic score. A prospective multicenter study should collect symptoms, infertility characteristics, examination findings, and IDEA-informed ultrasound features before surgery and without knowledge of laparoscopic findings. Such a design could test whether reproductive context adds clinically meaningful information to standardized imaging and could externally validate any resulting prediction model in an independent Congolese population.

## Conclusions

Among selected gynecologic laparoscopies performed in Kinshasa, superficial and ovarian endometriosis were frequently identified, and anatomical phenotype overlap was common. Of women with superficial peritoneal lesions and classifiable preoperative records, a substantial proportion lacked documented suspicion of endometriosis of any phenotype, most frequently when superficial disease was isolated. Associations with infertility and deep dyspareunia warrant cautious interpretation because documentation, referral pathways, and coexisting operative phenotypes could affect the findings. Prospective studies using standardized clinical recording and dedicated preoperative ultrasound assessment are required to investigate recognition of superficial lesions specifically.
